# Signaling by Steroid Hormones in the 3D Nuclear Space

**DOI:** 10.3390/ijms19020306

**Published:** 2018-01-23

**Authors:** François Le Dily, Miguel Beato

**Affiliations:** 1 Gene Regulation, Stem Cells and Cancer Program, Centre for Genomic Regulation (CRG), The Barcelona Institute of Science and Technology (BIST), Doctor Aiguader 88, 08003 Barcelona, Spain; francois.ledily@crg.es; 2 Universitat Pompeu Fabra (UPF), 08003 Barcelona, Spain

**Keywords:** chromatin conformation, estrogen receptor, steroid receptors, topological domains, transcription regulation

## Abstract

Initial studies showed that ligand-activated hormone receptors act by binding to the proximal promoters of individual target genes. Genome-wide studies have now revealed that regulation of transcription by steroid hormones mainly depends on binding of the receptors to distal regulatory elements. Those distal elements, either enhancers or silencers, act on the regulation of target genes by chromatin looping to the gene promoters. In the nucleus, this level of chromatin folding is integrated within dynamic higher orders of genome structures, which are organized in a non-random fashion. Terminally differentiated cells exhibit a tissue-specific three-dimensional (3D) organization of the genome that favors or restrains the activity of transcription factors and modulates the function of steroid hormone receptors, which are transiently activated upon hormone exposure. Conversely, integration of the hormones signal may require modifications of the 3D organization to allow appropriate transcriptional outcomes. In this review, we summarize the main levels of organization of the genome, review how they can modulate the response to steroids in a cell specific manner and discuss the role of receptors in shaping and rewiring the structure in response to hormone. Taking into account the dynamics of 3D genome organization will contribute to a better understanding of the pleiotropic effects of steroid hormones in normal and cancer cells.

## 1. Introduction

Similarly to other steroids, Estrogens (e.g., Estradiol, E2) exert their action by binding to their cognate nuclear receptors, the Estrogen Receptor (ER), which mainly functions as ligand-activated transcription factor [1,2]. Upon activation, ER translocates to the nucleus and converges to chromatin together with effectors of signaling pathways activated at the plasma membrane through non-genomic pathways [3]. ER binds directly to DNA through Estrogen Responsive Elements (ERE), which correspond to palindromic repeats [4], as well as indirectly through protein-protein interactions with other transcription factors [5,6]. It has been initially proposed that the effects E2 exerts on transcription depend on the binding of the ER to response elements located within the proximal promoters of the target genes. There, activated receptors orchestrate the recruitment of co-regulators, chromatin remodeling complexes and general transcription factors [1,2,7]. Although such mechanisms have been described in details for model estrogen responsive genes [8,9], the emergence of high-throughput technologies challenged this view: genome-wide analysis of transcripts levels by micro-arrays or RNA-Seq showed that the hormone modulate the expression of several hundreds of targets genes, many without direct binding of the ER at the proximal promoter [10,11,12]. Indeed, ChIP-Seq experiments targeting the ER in model estrogen-responsive cells showed that the receptors bind to DNA in an unexpected genome-wide fashion. The majority of the binding sites are located in intergenic regions, frequently far away from genes, and rather correspond to enhancer regions [13,14,15]. Similar observations have been made for other steroid receptors, such as the Glucocorticoid (GR) and Progesterone (PR) receptors, suggesting a shared mode of action [16,17]. 

Enhancers classically regulate transcription through chromatin looping to bring the regulatory machinery in close proximity to the promoters they target [18]. This level of chromatin folding and the formation of regulatory loops is embedded in more complex levels of organization of the genome. Indeed, it is becoming evident that the genome is organized in a highly compartmentalized and non-random fashion in the nucleus in interphase [19,20,21]. Such three-dimensional (3D) structures, in part cell specific, constrain the treatment of the genetic information in processes such as replication or transcription [22,23,24,25].

In terminally differentiated cells, 3D genome organization may constitute an epigenetic level controlling signal-induced modifications of transcription, as in the case of the rapid response induced by steroid hormones [26,27,28,29]. In the context of this review, we give an overview of the recent advances on understanding genome folding in eukaryotic cells and describe how it can interfere with the activity of transcription factors and the response to external cues. We further discuss observations of steroid dependent reorganization of the 3D genome architecture at local or more global scales.

## 2. Genome 3D Organization

Increasing experimental evidences support of a highly compartmentalized organization of the genome within the nucleus in interphase. Both cytological approaches such as Fluorescent In Situ Hybridization (FISH), or biochemical methods deriving from the chromosome conformation capture (3C) technique [30], have demonstrated that chromosomes do not decondense in a random way but rather organize following hierarchical order of structures [19,20,21]. The emergence of high-throughput 3C-derivatives, in particular Hi-C [31], allowed analysis of genome organization at various scales: individual chromosomes are organized in chromosome territories and are segmented in domains of preferential local contacts known as Topologically Associating Domains (TADs), which belong to functionally and epigenetically distinct chromatin compartments [24,25,31,32,33].

### 2.1. Chromosome Territories

**Figure 1:**
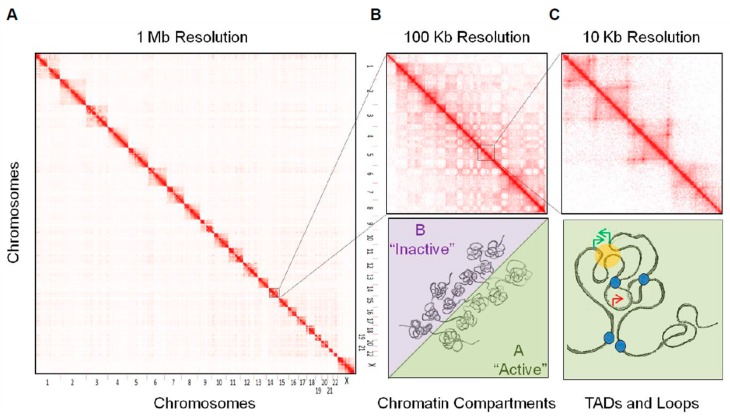
Hierarchical organization of the genome. Hi-C permits genome-wide detection of pair-wise contacts between genomic loci. They could be summarized as contact matrices where the color scale highlights the frequency of ligations events observed between any pairs of loci in the genome (from white to red, low to high frequencies, respectively). At different scales of resolution (e.g., 1 Mb, 100 or 10 Kb), higher orders of structure emerge: (**A**) chromosome territories, (**B**) chromatin compartments, (**C**) Topologically Associating Domains (TADs) and loops. (**B**,**C**) Bottom panels correspond to possible interpretations of the contact matrices: (**B**) active and inactive chromatin segregate spatially in two distinct chromatin compartments (A and B, respectively). (**C**) Architectural proteins (blue circles), such as CTCF (CCCTC-binding Factor), participate in the partitioning of the genome in TADs and generate sub-megabase structures, which can bring together specific loci or exclude genes from the activity of distal regulatory regions (orange circle: active enhancer; green arrows: expressed genes; red arrows: silenced genes).

The use of fluorescent whole chromosome paint probes permitted to confirm the hypothesis that chromosomes do not decompact in an unorganized way after mitosis but rather occupy a discrete area within the nuclear space [34,35]. These structures, known as chromosome territories, show limited intermingling between them and appear to distribute at preferential positions within the nucleus. In human cells, for example, long gene poor chromosomes are preferentially observed at the periphery, close to the nuclear lamina, while small gene rich chromosomes are frequently located within the central part of the nucleus. This suggests a functional radial positioning of chromosomes in relation to their transcriptional activity [35,36]. If the chromosome territories were originally observed by cytological approaches, this level of structure has also been confirmed by Hi-C experiments, which showed that most of the contacts detected were occurring in *cis* (i.e., intra-chromosomal contacts—Figure 1A) and that the *trans*, inter-chromosomal, interactions were reflecting the relative positioning of chromosomes [31].

### 2.2. Chromatin Compartments

In addition to support the existence of territories, the contact matrices obtained by Hi-C show a striking “plaid” or “chess” pattern (Figure 1B), which corresponds to the engagement of preferential long-range associations by non-contiguous genomic domains [31]. Such arrangement reflects the segregation of two types of genomic domains, which tend to not intermingle between them. Correlation of this pattern with epigenetic marks and transcription data demonstrated that the two types of regions corresponded mainly to the active and inactive parts of the genome (Figure 1B), also referred to as A and B chromatin compartments, respectively [31]. The existence of these two spatially segregated chromatin compartments has been confirmed by high- resolution microscopy using specific oligo-paint FISH probes [37]. This approach allowed the distinguishing of the two compartments spatially polarized in single chromosomes and further suggested that compartmentalization differs with the transcriptional activity [37]. This bimodal chromatin compartmentalization was initially observed based on Hi-C contact maps at resolutions between 0.1 to 1Mb. Recent high coverage Hi-C studies further demonstrated that the segregation of chromatin domains could be defined in a finer way, with the A and B compartments being subdivided in sub-compartments in correlation with their activity [33,38]. Importantly, this spatial segregation of chromatin compartments appears largely cell specific. Through the process of differentiation, chromosomal domains can dynamically switch from one to the other compartment, in correlation with changes of expression of tissue specific genes [23,39]. Conversely, in the process of dedifferentiation or cell reprogramming, chromosomal domains can change dynamically their association to one or the other compartment, in some cases prior to corresponding transcriptional modifications, supporting an instructive role of the 3D structure on transcription [40].

### 2.3. Topologically Associating Domains

At a resolution of 100 Kb or below, Hi-C chromosomal contact maps show that chromosomes are segmented in domains of high local interactions separated from each other by sharp boundaries (Figure 1C). These megabase-sized domains were referred to as Topological Domains, Topologically Associating Domains or TADs [24,32]. The boundaries between TADs are characterized by the presence of highly expressed housekeeping genes as well as by enrichments in epigenetic marks (e.g., H3K4me3, H3K36me3) linked to gene activation and in binding sites for architectural proteins such as CCCTC-binding Factor (CTCF) and cohesins [24,25,41,42]. In contrast to what is observed at the level of chromatin compartments, boundaries between TADs are largely conserved between cell types and through evolution. This suggests that TADs are important structural levels of organization [23,24]. However, a recent study based on a multi-scale analysis of insulation between genomic domains suggested that, rather to be a structurally favored level of organization, TADs represent an optimal functional level of folding for the establishment of specific interactions [43]. Such organization will notably facilitate the coordinated regulation of genes by facilitating the organization of specific wiring between genes promoters and regulatory elements [43]. In this view, TADs can be considered as epigenetic domains characterized by relatively homogeneous epigenetic features, suggesting that the border between them could limit the spreading of epigenetic marks [24,25,32]. In addition, TADs can behave as transcription units where genes are co-regulated under the control of specific regulatory elements during differentiation [32,43,44] or in response to steroid hormones [28].

### 2.4. Sub-Domains and Chromatin Loops

If the boundaries between TADs are conserved between cell types, their internal organization appears more dynamic and cell specific. At higher resolutions, the contact maps show cell specific internal sub-TADs or sub-domains (Figure 1C), which correspond to structures generated by the interactions between specific elements, either structural and/or related to gene activity [33,38,45,46]. In particular, during differentiation, differential binding of CTCF and cohesins, together with subunits of the mediator complex lead to the formation of such cell specific sub-domains [45]. This sub-megabase level of organization leads some authors to propose that chromosome-neighborhoods, which correspond to the establishment of specific CTCF loops that embed genes together with or without regulatory elements, might be the functional minimal unit of organization of the genome [33,38,47]. Additionally, other zinc finger proteins able to form homodimers, such as YY1, can mediate enhancer-promoter loops and are essential for specific gene regulation [48]. 

In summary, the genome is organized in a hierarchy of structures that have been correlated with the processing of the genetic information. Although these preferential structures can be observed in cell populations, it is important to keep in mind that, in single cells, the underlying spatial interactions remain highly dynamic and rather stochastic, as exemplified by results obtained in single cell Hi-C [49] and by the frequent discrepancies observed between 3C derived population results and direct visualization in single cells by FISH [50]. Globally however, genome-wide contact datasets suggest a cell type specific organization, which could participate in the integration of the different signals received by the cell. Degron-mediated knock-down of the levels of CTCF or subunits of the cohesin complex lead to a loss of the organization in loops and TADs without affecting the segregation of chromatin compartments [51,52,53]. These studies confirmed that architectural proteins are essential for the maintenance of cell specific organization of TADs and suggest that the different levels of structure are partially uncoupled. However, these proteins probably act in combinations with other regulatory factors but the precise role of tissue-specific transcription factors in organizing different levels of organization remains largely unexplored.

## 3. 3D Genome Folding Modulates the Response to Steroids

Comparative ChiP-Seq studies demonstrated that the landscape of binding of steroid receptors varies quantitatively and qualitatively from cell type to cell type, even between cells lines of similar origins [17,54]. This cell specificity probably participates in the regulation of distinct subsets of responsive genes, explaining the differences observed in different cell lines in response to the same stimulus [55,56]. The different levels of structures described above are part of the cell identity and one can reasonably hypothesize that they act as an epigenetic level to condition the activity of transcription factors. In particular, in the case of steroid receptors, which activity is regulated by external signals in terminally differentiated cells, this 3D organization can participate in demarcating the sets of regulatory elements potentially bound by the receptors as well as in restricting the genes that will be targeted.

### 3.1. Steroid Receptors Cistrome

Although variables depending on cell types, time of treatment and detection approaches, results from transcriptomic studies showed that steroid hormones elicit genome-wide changes in gene expression, with between hundreds to thousands of genes being either up- or down-regulated upon hours of treatment [10,11,12,16]. Some of these changes rely on indirect secondary regulations; nevertheless, the use of Global-Run-On method (GRO-Seq) confirmed these broad effects of E2 on transcription [12]. In addition, GRO-Seq permitted to highlight rapid changes in transcription not only of protein coding genes but also of many non-annotated, non-coding transcripts [12]. Rather than giving a direct explanation to these broad transcriptional changes, analysis of ER binding by ChIP-on-chip or later on by ChIP-Seq experiments largely modified the classical view of the mechanisms involved in the cellular response to steroids [13,15,57]. For instance, ChiP-Seq experiments performed in MCF-7 cells after treatment with E2 demonstrated an unexpected genome-wide binding of the ER, with more than 14,000 binding sites detected; much more than the number of genes actually regulated by the hormone in these cells [15]. The location of these binding sites throughout the genome was also unexpected: only a small proportion was located within the proximal regulatory regions of targets genes; the majority of sites were rather broadly distributed, with particular enrichment in distal inter-genic regions. This suggests that, in addition to act at the levels of promoters, ER exert their actions from distal regulatory regions. Similar behaviors were observed in other cell types and for other nuclear receptors, such as the GR and the PR [16,17]. For instance, upon 30 min exposure to progestins, PR bind to more than 25,000 sites characterized by enhancer marks and located at more than 30 Kb away from promoters [16]. This suggests a shared mode of actions of steroid receptors in acting as regulators of the activity of distal, enhancer or silencer, regions.

### 3.2. Differential Accessibility of Hormone Response Elements

Despite the large number of steroid receptors binding sites detected by ChIP-Seq, they represent only a small fraction of the potential Hormone Response Elements (HRE) identified by searching for consensus sequences in the genome [58], suggesting that a large fraction of HRE is not accessible for binding. This differential accessibility represents the first level of epigenetic regulation of steroid receptors function as transcription factors and can consequently dictates the cell specific target genes. Several mechanisms can explain why the receptors can only bind to part of their consensus element on DNA. ER, for example, binds preferentially to DNA elements that are not protected by nucleosomes, and requires the activity of pioneer factors. Pioneer factors are able to bind to nucleosomal DNA where they orchestrate local arrangements of the chromatin and therefore act cooperatively to facilitate the binding of activated receptors [59]. For example, Forkhead Box Protein A1 (FOXA1) has been shown to mark the sites that will be bound by ER after exposure to the hormone [60]. The PR is able to bind to sites occupied by nucleosomes [16] and other mechanisms, such as methylation of DNA, potentially prevent its binding to some of its responsive elements [61].

**Figure 2:**
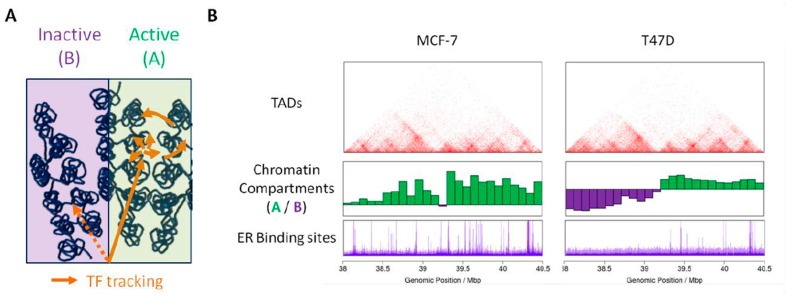
3D genome folding modulates the binding of steroid receptors. (**A**) Active and inactive chromatin compartments can favor or limit, respectively, the diffusion of transcription factors within the nuclear space. Long-range folding of the chromatin fiber can facilitate the tracking and permit local enrichment of factors in given nuclear environments. (**B**) In contrast to the conservation of borders between TADs (top panel—frequencies of contacts in red), chromosomal domains belong to the A (green) or B (purple) compartment in a cell specific manner (middle panel). These differences correlate with the extent of binding of the ER as shown by ChIP-Seq experiments in MCF-7 and T47D (bottom panel).

More generally, the precise mechanisms by which transcription factors find their DNA targets within the nuclear environment remains mainly unknown [62]. In addition to epigenetic modifications of DNA and nucleosomes, the way the chromatin is spatially organized in the nucleus can interfere with, either promote or limit, the accessibility of chromatin to nucleoplasmique factors. Several models of transcription factor search strategies have been proposed, which notably include facilitated diffusion through chromatin. In this model, transcription factors binding to specific chromatin sites occurs through a combination of diffusion, linear tracking and jumps, or hopping, along the chromatin fiber [63]. The fact that the genome folds readily in a non-random manner may favor such hopping from regions far away on the linear genome and may direct transcription factors to specific sites (Figure 2A). In addition, pre-existing chromatin loops can favor the residence time of transcription factors and lead to the nucleation of transcription factors enriched environments [64]. Finally, active and inactive (A/B) chromatin compartments are characterized by epigenetic signatures associated with open or compact chromatin, respectively. Transcription factors may therefore preferentially access (and bind to) the accessible active chromatin compartment (Figure 2A). The fact that domains belonging to the same compartment are found preferentially together within the nuclear space can delimit the space that transcription factors have to visit prior to reach functional sites. In this context, the cell specific compartmentalization of chromatin can contribute to the establishment of a cell specific landscape for binding of transcription factors. In line with this hypothesis, differential binding of the ER in the breast cancer cells MCF-7 and T47D is observed in genomic domains that belong to distinct chromatin compartments (Figure 2B).

### 3.3. Topological Restraint of Promoter-Enhancer Looping

**Figure 3:**
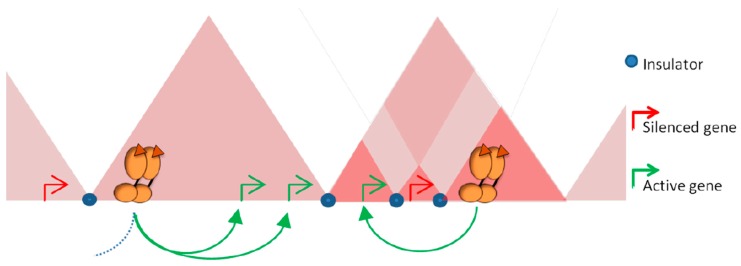
Structural segmentation of the genome restrains the range of action of regulatory elements. The enrichment in proteins with insulator function at the borders between TADs can prevent contact with regulatory elements activated by steroid receptors with promoters located outside of the domains. The natural tendency of contacts within a TAD limits the space to be explored by an activated enhancer and favor the stability of promoter-enhancer contacts within the domain independently of the genomic distance. Intra-domain loops established in a cell specific manner can serve to isolate genes from the activity of distal regulatory elements or conversely, favor contacts between enhancers and specific sets of genes.

An important, still opened, question is to determine whether all the sites detected by ChIP-Seq are functional and participate in transcriptional regulation or whether they are reflecting non-productive binding or chromatin interactions involved in other processes. The use of ChIA-PET, a method allowing the analysis of the spatial contacts between loci bound by a specific factor [65], showed that many of the ER bound loci were interacting together to form complex loops anchoring distal and proximal ER sites [27]. These observations support the concept that the inter-genic ER binding sites correspond to enhancer or silencer regions, which could act on distal target genes by chromatin looping. In this context, higher levels of organization may constrain the activity of the receptors by delimiting the targets they can reach. Most of the interactions between enhancers and promoters occur within TADs [47,66,67]. By limiting the space to be explored, this level of organization favors the activity of regulatory elements on the genes lying within the same domain, independently of the genomic distance that separate them (Figure 3). Conversely, the boundaries that separate contiguous domains can act as barriers and impede ectopic action of regulatory elements on genes located outside of the domain (Figure 3). The importance of these boundaries in demarcating the targets of a given regulatory region is supported by experiments where the borders between TADs were specifically deleted [68]. The use of CRISR-Cas9 approach to engineer borders between contiguous TADs induces structural modifications, with establishment of novel interactions between enhancers and promoters. This rewiring is accompanied with a misexpression of the genes located outside of the original domain, supporting a role for TADs in demarcating the range of action of regulatory elements [68]. Within a given TAD, additional cell specific loops can generate sub-domains, or chromosome neighborhoods [33,38,45,47]. These local structures can serve to isolate given genes from the activity of regulatory elements in order to maintain them in a silenced state (Figure 3) or conversely to favor the regulation of a given set of genes [47]. 

The organization of the genome in chromosomal domains is therefore both limiting the activity of given regulatory elements to a specific set of target genes and demarcating the 3D space that has to be explored by a regulatory element to engage specific looping with its targets. This can explain the frequent genomic clustering of steroid responsive genes as well as their co-regulation within TADs in response to hormones [28,69,70,71].

### 3.4. Influence of Architectural Proteins in Steroid Response

There are evidences that architectural proteins, notably CTCF and cohesins, are directly involved in the response to E2 [72,73]. Depletion of CTCF affects the transcriptional response to E2 of model responsive genes [74]. ER binding sites frequently co-localize with CTCF sites in a cell specific manner, suggesting that CTCF can direct the ER to specific regions, potentially acting upstream of FOXA1 or other pioneer factors [73]. ER is also found at sites occupied by cohesins in a CTCF independent manner and those sites are associated with responsive genes and more prone to establish chromatin loops [75]. In this line, depletion of CTCF or RAD21 Cohesin Complex Component by small interfering RNA (siRNA) led to destabilization of the loops engaged by ER binding sites [74]. In line with the models described above, those architectural proteins can participate in licensing ER binding in a cell specific manner and/or can facilitate the establishment of specific regulatory chromatin loops. In link with its function as insulator, CTCF also demarcates specific chromosome neighborhoods [47] or regulatory units, which could restrain the range of interactions of a given ER bound site [76].

## 4. Steroid Receptors Mediated Genome (re)-Organization

As described above, the conformation of the genome can restrain the response to steroid hormones by modulating the binding of the receptors to chromatin as well as by demarcating the target genes that could be regulated in a given cell type. Once bound to their DNA elements, the receptors recruit a plethora of co-factors, which act as nucleosomes remodeling machinery or histones modifying enzymes [7,77,78,79]. Recruitment of these co-regulators leads to either local or long-range modifications of the chromatin fiber, which can consequently reshape the 3D organization and may be essential to set the stage for subsequently acting transcription regulators.

### 4.1. Steroid Receptors Dependent Promoter-Enhancer Loops

The existence of functional chromatin loops between distal steroid receptor binding sites and promoters during the activation of model genes, such as *TFF1* and *CTSD*, have been demonstrated by 3C [27,74,80]. In addition to loops between enhancer and promoters, there are also evidences for steroid induced looping involved in gene repression [81]. These experiments, performed in absence or presence of ligand, showed increased frequency of contacts of the distal regions with proximal promoters upon binding of the receptors at the regulatory sites, suggesting that the binding of the receptors has an instructive role on the looping. However, how receptors actually reorganize the folding of these loci remains mainly hypothetical. Remodeling of nucleosomes as well as modifications of histones tails, at or around receptors binding sites, lead to an opening of chromatin. This could provide more flexibility to the chromatin fiber, increasing the probability for the looping to occur. In addition, ER and other steroid receptors are known to interact with components of the mediator complex, such as the Mediator Complex Subunit 1 (MED1), which could help in the stabilization of the contacts between distal transcription machineries [82,83,84]. It has also been observed that enhancers bound by ER are sites of transcription of short RNA products known as enhancer RNA (eRNA)—[12,85,86]. These RNAs may participate in the stabilization of the contacts between distal elements and/or in the recruitment of additional co-factors [87]. However, it is still not clear whether their production is necessary and sufficient to induce the looping between enhancers and target promoters. Inhibition of the production of the eRNA did not prevent the formation of loops between enhancer and promoter when MCF-7 cells were co-treated with E2 and flavopiridol [86]. In other models, the expression of non-coding RNA was necessary to the establishment of productive looping [87]. 

### 4.2. Dynamic and/or Pre-Settled Organization of Steroid Responsive Hubs

**Figure 4:**
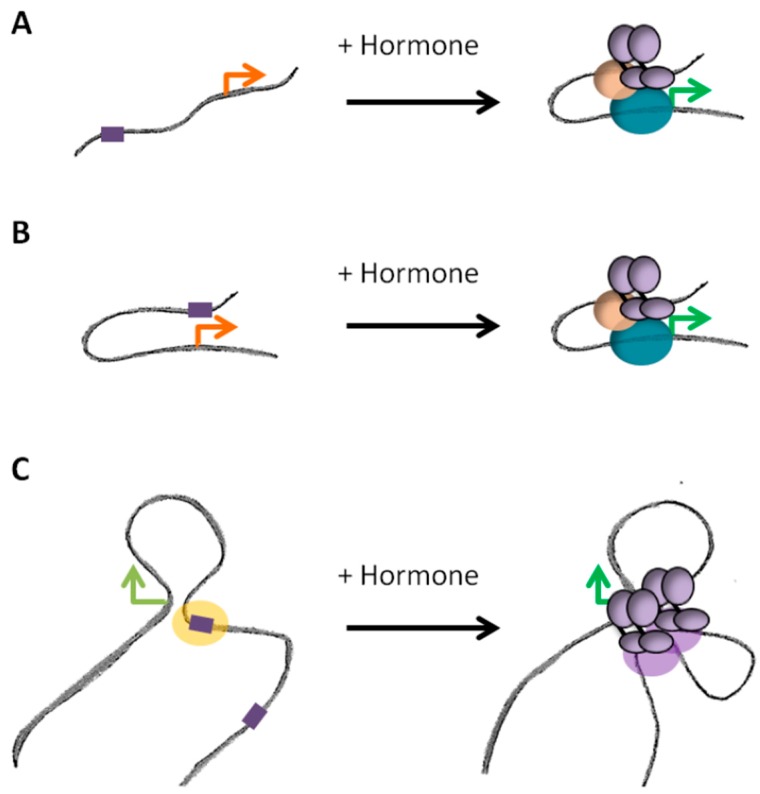
Different dynamics of promoter-enhancer looping. (**A**) Binding of the receptors upon exposure to the hormone induces the formation of loop between promoter and enhancer in an active process. (**B**) In other cases, the loop is established prior binding of the receptors, which activate the enhancer region in an already favorable conformation. (**C**) A promoter could be regulated by several enhancers, which require or not de novo chromatin looping. Binding of steroid receptors in the absence of hormones (unliganded receptors) might serve in maintaining such structures prior exposure to the hormone. Orange and green arrows correspond to paused or activated promoters, respectively.

Genome-wide studies of chromosome conformation highlighted that contacts between enhancers and promoters occur in a complex fashion: enhancers frequently contact multiple targets and a given gene can be submitted to the activity of several enhancers [67]. The existence of such chromatin hubs is supported by ChIA-PET data where ER binding sites were observed to be engaged in multiple interactions, generating a complex network of loops and anchors after exposure to hormone [27]. Establishment of loops between multiple enhancers and promoters was observed for other nuclear receptors [26,28,88]. It remains unclear however whether those hubs are relatively stable structures or whether they are established de novo upon binding of the activated receptors (Figure 4). ChIA-PET per se does not permit to determine whether the contacts observed depend or not on the binding of the receptors. However, in the same study, various alternative approaches (3C, 4C and FISH) were used to confirm that the looping network was dependent on hormone exposure [27]. In response to progestins, changes in transcription are associated with concomitant changes in chromatin structure and conformation of TADs [28], arguing for hormone induced rewiring of local spatial contacts (Figure 4A). On another hand, some contacts between poised enhancers and promoters have also been observed prior to exposure to the signal (Figure 4B). In this case, activated receptors bind within a pre-established structure and the binding does not dramatically modify the existing contacts [46]. Steroid receptors themselves could participate in maintaining those pre-existing loops in the absence of hormones. Indeed, large regulatory regions of clustering of ER and PR after exposure to the hormones are frequently already occupied by the unliganded receptors in basal conditions. The structure of TADs exhibiting binding of unliganded ER or PR within these regions largely differ between cells expressing or not the receptors, suggesting a direct role for the unliganded receptors in maintaining a structure that could facilitate further binding of the receptors after activation [88]. Active looping induced by the hormones or binding within pre-existing structures are probably not mutually exclusive models of action (Figure 4C). For instance, in the case of the response to glucocorticoids, the use of ChIA-PET targeting p300 showed that a large fraction of enhancers bound by the GR upon exposure to the hormone were already bound by p300 and engaged in interactions with gene promoters prior to exposure to the hormone. In parallel, GR also brought de novo the histone acetyl-transferase to a significant fraction of sites, which interactions with promoters and pre-existing sites were dynamically modified by the hormone [26].

### 4.3. Steroid Induced Changes at Higher Levels of Organization

As mentioned above the loops between regulatory elements and promoters appear to be limited to loci laying in the same TAD and exposure to steroid hormones can lead to local restructuration of TADs, reflecting a rewiring of the contacts [26,27,28]. Some observations also suggest that steroid responsive loci located further away are found together within the nuclear space. For instance, clusters of binding sites of ER and PR located in different TADs can establish long-range interactions between them [88] and genes responsive to glucocorticoids have been observed to cluster within nuclear hubs [89]. These observations could suggest the existence of specific hubs or transcription factories specialized in the response to steroids. In addition, steroids have been shown to induce large-scale remodeling of chromatin [90,91]. This can occur through spreading of chromatin modifications over large chromatin domains or even more globally within the nucleus [28,81,92]. Whether these large-scale modifications are accompanied with more global changes of the 3D genome structure remains unclear. Despite the important changes of chromatin induced by progestins, dynamic changes of chromosomal organization were mainly observed at the level of TADs [28]. Similarly, the long-range clustering of glucocorticoid responsive genes in nuclear hubs was not modified upon exposure to the hormones [89]. In contrast, exposure to E2 have been proposed to induce large-scale reorganization of the structure of chromosomes [93] and some authors proposed that E2-responsive genes could be actively brought together within the nuclear space upon exposure to the hormone, facilitating their transcription in specific hubs [94]. However, such active clustering of E2-responsive genes was not observed by others in similar models [95].

Although such long-range organizations may be favored, they probably remain highly stochastic and it will be important to determine to what extent they facilitate the transcriptional response. Additional studies of their dynamic will be also necessary to determine to what extent they are reflected by functional modifications of higher levels of structure of the genome.

## 5. Future Directions

In summary, the way the genome organizes can modulate the activity of steroid receptors and other transcription factors at several levels: accessibility to their binding sites, topological restraint of their potential targets as well as facilitation of effective enhancer/promoter looping. Conversely, the modifications that the receptors exert on chromatin upon exposure to their cognate signal lead to specific restructuration of the chromatin folding, which are probably important for fine-tuning the transcriptional response. Together these observations support a role for steroid receptors as genome organizers, not only at local but also at global scale.

In addition to their direct effects on chromatin, it is also important to consider that steroids exert important so-called non-genomic actions, which can actively participate in the final transcriptional output [96,97]. For instance, estrogens and progestins can rapidly activate protein kinases [96,97,98,99] and chromatin effectors such as Poly(ADP-Ribose) Polymerase 1 (PARP1) [100] that are both important for their transcriptional effects. Activation of PARP1 upon exposure to hormones leads to the formation of Poly-ADP-ribose (PAR), which directly acts on chromatin structure through histone H1 displacement [100] as well as on the synthesis of nuclear ATP required for the response to estrogen and progestins [101]. Interestingly, PAR has been associated with phase transition mechanisms [102] and ATP acts as an hydrotrope for liquid droplet formation [103]. Such processes have been recently highlighted for their potential role in the distribution of proteins and chromatin within the nuclear space. More profound analysis of these non-genomic processes and their consequences on genome structure will be important to better understand the pleiotropic effects that steroid hormones have on gene transcription and cell fate. 

Finally, if the 3D structure of the genome plays a role in transcription regulation in normal cells, one could easily hypothesize that its modifications may favor inappropriate responses. Indeed, chromosomal rearrangements could have dramatic influence on gene expression by modifying the normal landscape of action of enhancers [68,104]. Juxtaposition of specific chromosomes or genomic domains in the nucleus in normal cells reflects the preferential breakpoints that lead to oncogenic fusion proteins in some cancers [105]. Hormone induced double strand breaks upon binding of the Androgen Receptor (AR) has been involved in such processes in prostate cancers [106]. A better understanding of how these events occur is of particular importance in diseases associated with response to steroid hormones, such as breast or ovarian cancers, where the genome is frequently rearranged during the process of transformation.
